# Rural–Urban Differences in Behavioral Outcomes among Adults with Lifetime History of Traumatic Brain Injury with Loss of Consciousness: 2016–2019 Ohio BRFSS

**DOI:** 10.3390/ijerph19031678

**Published:** 2022-02-01

**Authors:** Robyn Feiss, John D. Corrigan, Kele Ding, Cynthia L. Beaulieu, Jennifer Bogner, Jingzhen Yang

**Affiliations:** 1 Center for Injury Research and Policy, The Abigail Wexner Research Institute at Nationwide Children’s Hospital, Columbus, OH 43215, USA; robyn.feiss@nationwidechildrens.org; 2 Division of Rehabilitation Psychology, Department of Physical Medicine and Rehabilitation, College of Medicine, The Ohio State University, Columbus, OH 43210, USA; johncorrigan1@me.com (J.D.C.); Cynthia.Beaulieu@osumc.edu (C.L.B.); Jennifer.Bogner@osumc.edu (J.B.); 3 Department of Health Sciences, Kent State University, Kent, OH 44240, USA; kding@kent.edu; 4 Department of Pediatrics, College of Medicine, The Ohio State University, Columbus, OH 43210, USA

**Keywords:** binge drinking, depression, heavy drinking, mental health, population-based

## Abstract

This study examined if the associations between lifetime history of traumatic brain injury (TBI) with loss of consciousness (LOC) and unhealthy alcohol use or mental health problems differ by location of living (rural vs. urban). The lifetime history data of TBI with LOC, location of living, unhealthy alcohol use (binge drinking, heavy drinking), and mental health problems (depression diagnosis, number of poor mental health days) were sourced from the 2016, 2017, 2018, and 2019 Ohio Behavioral Risk Factory Surveillance Surveys, and the final sample included 16,941 respondents. We conducted multivariable logistic regressions to determine the odds ratios for each of the five outcomes between individuals living in rural vs. urban areas and between individuals with vs. without a lifetime history of TBI with LOC. No interaction between location of living and lifetime history of TBI with LOC was observed for any outcomes, indicating rurality did not modify these relationships. Living in a rural area was associated with decreased binge drinking or heavy drinking but not mental health outcomes. Lifetime history of TBI with LOC was associated with an increased risk of binge drinking, heavy drinking, depression diagnoses, and poor general mental health, regardless of location of living. Our findings support the need for TBI screenings as part of mental health intake evaluations and behavioral health screenings. Though rurality was not associated with mental health outcomes, rural areas may have limited access to quality mental health care. Therefore, future research should address access to mental health services following TBI among rural residents.

## 1. Introduction

Traumatic brain injury (TBI), a significant public health issue in the US, resulted in over 2.87 million emergency department visits, hospitalizations, or deaths in 2014 [1], costing an estimated $86 billion annually [2]. Importantly, TBI, a neurological condition occurring when an external force alters normal brain function, either temporarily or permanently [3], can have both short- and long-term health consequences, including increased likelihood of behavioral health problems [4]. Given the association between TBI and long-term health consequences, research has begun to focus on lifetime history of TBI, particularly those TBIs with loss of consciousness (LOC), and associated behavioral health problems, such as unhealthy alcohol use and mental health problems [4,5].

Multiple statewide population-based studies have demonstrated that lifetime history of TBI with LOC is associated with unhealthy alcohol use [5,6] and mental health problems [5,6,7]. Bogner and colleagues found, among non-institutionalized adult Ohioans, having at least one TBI with LOC was associated with increased binge drinking, heavy drinking, a diagnosed depressive disorder, and having poor mental health for 2 or more and 14 or more days in the past 30 days. A study using data from North Carolina’s 2017 Behavioral Risk Factor Surveillance System (BRFSS) survey showed similar associations between lifetime history of TBI with LOC and alcohol use, binge drinking, and lifetime depression diagnoses [6]. An earlier population-based survey of adult Coloradoans found that severe TBI (i.e., TBI with LOC longer than 24 h) was associated with reporting 2 or more days of poor mental health but did not find a relationship between TBI of any severity and alcohol use [7]. While these and other studies have examined individual differences associated with the co-occurrence of TBI and unhealthy alcohol use, none have addressed the potential associations of rurality with these relationships.

The existing evidence suggests that both children [8] and adults [9,10] living in rural or remote areas are at a greater risk for TBI and have poorer health outcomes following TBI [9,11] than those living in urban or suburban areas. However, limited studies have addressed the relationships between lifetime history of TBI (with or without LOC) and location of living (e.g., rural vs. urban). One study in New South Wales demonstrated that rural patients were more likely to have a mental health illness following brain injury [12]. Furthermore, the rates of alcohol use and unhealthy alcohol use are generally lower among those living in rural areas compared to those living in urban areas [9,10], but it is not known if rurality continues to act as a protective factor against unhealthy alcohol use among those who have had a TBI with LOC who are at higher risk for alcohol use than those without such a history [5]. Finally, the prevalence of depression is higher among those living in rural areas compared to those living in urban areas [13], largely due to cultural, social, and economic factors associated with rural living, including greater rates of poverty and physical inactivity [13,14]. While factors including sex [14], gender [13], race [13,14,15], age [13,14], and other health characteristics [13] may contribute to the difference, it is unclear how rurality may be related to the mental health of adults with a lifetime history of TBI, who are at an increased risk for mental health problems [5].

Given the gaps in the current research on the associations of rurality with lifetime history of TBI and risk of unhealthy alcohol use and mental health problems in an adult population, this study aimed to determine if the relationships between lifetime history of TBI with LOC and unhealthy alcohol use or mental health problems differ by location of living (rural vs. urban). We expected that living in a rural area would be associated with a reduced risk of unhealthy alcohol use and increased risk of mental health problems, while having a lifetime history of TBI with LOC would be associated with an increased risk of unhealthy alcohol use and mental health problems.

## 2. Materials and Methods

### 2.1. Data and Study Participants

For this study, data were sourced from the 2016, 2017, 2018, and 2019 Ohio BRFSS [16] conducted among non-institutionalized adults aged 18 years or older. The BRFSS uses random-digit dialing to collect information annually on chronic conditions and health-related risk behaviors via a telephone survey [16]. During years 2016 to 2019, The Ohio BRFSS included lifetime history of TBI optional module, an adapted version of the Ohio State University TBI Identification Method (OSU TBI-ID) [17]. We included non-institutionalized adults ≥18 years who completed the core component questions and the lifetime history of TBI optional module of the 2016, 2017, 2018 and 2019 surveys in our sample (*n* = 19,896). Of these, a total of 2955 (14.9%) respondents were excluded from the analysis, including 410 (2.1%) respondents with missing values of location of living, 173 (0.9%) respondents aged < 18 years, and 2372 (11.9%) respondents with unknown information on ever being knocked out or losing consciousness from a reported head/neck injury. Thus, the final sample included 16,941 respondents. To ensure results were representative of the Ohio non-institutionalized adult population, the sample was reweighted. The final weight was calculated by multiplying the sample weight for each individual year by its original sample size proportion of the combined dataset (*n* = 19,896).

### 2.2. Variables and Measures

Lifetime history of TBI with LOC was defined as an injury to the head or neck that resulted in being knocked out or losing consciousness. The psychometrics and validation of the OSU TBI-ID are described in detail by Corrigan and colleagues 2020 [5]. This adapted version of the OSU TBI-ID includes 6 items to measure lifetime history of TBI with LOC. Individuals reporting at least one injury to the head or neck were further asked if they were ever knocked out or lost consciousness from their reported injury to determine lifetime history of TBI with LOC.

Location of living was categorized as rural (living in a non-metropolitan or non-micropolitan county in Ohio) or urban (living in a metropolitan or micropolitan county in Ohio). Metropolitan counties contain a core urban area of 50,000 or more and micropolitan counties contain a core urban area of 10,000 to 49,999 [18]. Counties were identified based on their Federal Information Processing Standards (FIPS) codes.

Unhealthy alcohol use was measured using two variables: (1) self-reported binge drinking in the past 30 days, defined as ≥5 drinks on one occasion for men or ≥4 for women, and (2) self-reported heavy drinking in the past 30 days, defined as >14 drinks per week for men and >7 drinks per week for women. A binary response category was used for each of these two variables [19].

Mental health variables included depression diagnoses and number of poor mental health days in the past 30 days. Specifically, for depression diagnosis, respondents were asked “Has a doctor, nurse, or other health professional ever told you that you had a depressive disorder, including depression, major depression, dysthymia, or minor depression?”. For number of poor mental health days, respondents were asked “Now thinking about your mental health, which includes stress, depression, and problems with emotions, for how many days during the past 30 days was your mental health not good?” [16] The number of poor mental health days was further categorized into two dichotomous variables: 2 or more days in the past 30 days (yes or no), and 14 or more days in the past 30 days (yes or no). This categorization allowed for comparison to other studies using BRFSS data [5,7,20], as well as comparison to standards of symptom severity commonly used for mental health diagnoses.

### 2.3. Statistical Analysis

We calculated the unweighted and weighted frequencies, and percentages of respondents along with their demographic characteristics. Additionally, we calculated weighted frequencies and percentages of respondents who reported binge drinking in the past 30 days, heavy drinking in the past 30 days, having a depressive disorder, having poor mental health days ≥ 2 in the past 30 days, and having poor mental health days ≥ 14 in the past 30 days. We conducted a stratified analysis to compare differences in unhealthy alcohol use, poor mental health outcomes, and co-occurrence of alcohol and mental health issues across subgroups among rural or urban respondents using χ2 tests. Finally, we conducted separate multivariable logistic regressions to determine the odds ratios (OR) for each of the behavioral outcomes (binge drinking, heavy drinking, depression diagnosis, and poor mental health days) between individuals living in rural areas vs. those living in urban areas and between individuals with vs. without lifetime history of TBI with LOC. The interaction between lifetime history of TBI with LOC and location of living was assessed in the regression analysis for each of the outcome variables. Since the interactions were not statistically significant for any of the outcome variables, the interaction term was not included in the final models. Variables adjusted for in the logistic regression models included gender, age group, and race/ethnicity. All model tests were also adjusted for sample stratum, cluster, and proportional weight variables. Statistical significance was set at α = 0.05 for all analyses, and all analyses were performed in SAS 9.4 (SAS Institute, Cary, NC, USA).

## 3. Results

Of the 16,941 survey participants included, 10,002 were female, 3596 were ages 18 to 44, 14,902 were White, and 10,285 were living in an urban area (Table 1). Due to the split assignment of the optional module containing the lifetime TBI questions, 7128 surveys were from 2016, which was about twice the number of surveys in each of the other three years. When weighted, it represented 7,578,787 non-institutionalized adult Ohioans during the four study years, including 52.1% females, 43.1% ages 18 to 44 years, 81.2% White, and 77.1% living in an urban area. Among the non-institutionalized adult Ohioans, 16.9% had experienced a TBI with LOC in their lifetime.

Overall, 16.9% of all the respondents reported binge drinking, 6.6% reported heavy drinking, 20.1% reported a diagnosed depressive disorder, 33.4% reported having poor mental health for 2 or more days of the past 30 days, and 14.2% reported poor mental health for 14 or more days (Table 2). Of the participants living in rural areas, lifetime history of TBI with LOC was associated with increased proportions of binge drinking (*p* < 0.001), heavy drinking (*p* < 0.001), depressive disorders (*p* < 0.001), poor mental health for 2 or more days (*p* < 0.001), and poor mental health for 14 or more days (*p* < 0.001). Similar findings were observed among those living in urban areas. Additionally, males living in rural areas were more likely to report binge drinking (*p* < 0.001) and heavy drinking (*p* < 0.001) than females living in rural areas, although females living in rural areas were more likely to report depressive disorders (*p* < 0.001), poor mental health for 2 or more days (*p* < 0.001), or 14 or more days (*p* < 0.001) than males. Again, similar results were observed among the respondents living in urban areas (Table 2). Lastly, among the rural respondents, racial differences were observed for depressive disorders, with non-White respondents and respondents of other races more likely to have depression diagnoses than White respondents (*p* = 0.02). This difference was not evident among the urban respondents, for whom there were no racial differences in reported depressive disorders. Additionally, among urban Ohioans, those with unknown race were at the greatest risk for binge drinking (*p* < 0.01) and heavy drinking (*p* < 0.01), and respondents of other races were the most likely to report having poor mental health for 2 or more (*p* < 0.05) or 14 or more days (*p* < 0.05) (Table 2).

Of those living in rural areas and who reported heavy drinking, 20.6% reported a co-occurring depression diagnosis, 35.4% reported 2 or more poor mental health days, and 19.3% reported 14 or more poor mental health days. The rates of co-occurrence did not differ from their urban counterparts, who reported depression diagnoses (23.1%, *p* = 0.38), 2 or more poor mental health days (36.2%, *p* = 0.83), and 14 or more poor mental health days (16.6%, *p* = 0.30), respectively. Furthermore, among the respondents living in rural areas who reported binge drinking, 19.9% reported a co-occurring depression diagnosis, 35.8% reported 2 or more poor mental health days, and 16.3% reported 14 or more poor mental health days. Again, co-occurrence did not differ between the rural and urban groups, with 21.3% (*p* = 0.44), 37.7% (*p* = 0.37), and 14.9% (*p* = 0.40) of urban heavy drinkers reporting depression diagnoses, 2 or more poor mental health days, and 14 or more mental health days, respectively.

No interaction between lifetime history of TBI with LOC and location of living was found. After adjusting for gender, age group, and race and location of living, those with lifetime history of TBI with LOC were 1.36 times (AOR = 1.36; 95%CI = 1.14–1.61) more likely to report binge drinking and 1.52 times (AOR = 1.52; 95%CI = 1.20–1.91) more likely to report heavy drinking than those without lifetime history of TBI with LOC (Table 3). Ohioans living in rural areas were less likely to report binge drinking (AOR = 0.85; 95%CI = 0.73–0.97) or heavy drinking (AOR = 0.75; 95%CI = 0.62–0.92) compared to those living in urban areas after adjusting for lifetime history of TBI status. Furthermore, those with lifetime history of TBI with LOC were 2.61 times (AOR = 2.61; 95%CI = 2.26–3.01) more likely to be diagnosed with a depressive disorder, 1.96 times (AOR = 1.96; 95%CI = 1.72–2.24) more likely to report poor mental health for 2 or more days, and 2.68 times (AOR = 2.68; 95%CI = 2.28–3.16) more likely to report poor mental health for 14 or more days compared to those without lifetime history of TBI with LOC (Table 4). Living in a rural area, however, was not associated with an increased risk for diagnosis of a depressive disorder (AOR = 0.98; 95%CI = 0.87–1.10) or having poor mental health for 2 or more (AOR = 0.93; 95%CI = 0.84–1.04) or 14 or more (AOR = 1.03; 95%CI = 0.89–1.19) of the past 30 days compared to living in an urban area after adjusting for lifetime history of TBI status (see Table 4). Similar findings were observed before adjustment for demographic characteristics and lifetime history of TBI.

Females and non-Whites were less likely to report binge drinking or heavy drinking compared to males and Whites, respectively (Table 3). Though race was not associated with the likelihood of mental health problems, females were 1.99 times more likely to be diagnosed with a depressive disorder, 1.68 times more likely to report poor mental health for 2 or more days, and 1.57 times more likely to report poor mental health for 14 or more days compared to males (Table 4).

## 4. Discussion

This study sought to determine if the relationships between lifetime history of TBI with LOC and unhealthy alcohol use or mental health problems differ by location of living (rural vs. urban). Our results showed that no interaction between location of living and lifetime history of TBI was observed for any outcomes. Further, non-institutionalized adult Ohioans living in rural areas were less likely to report binge drinking or heavy drinking but were just as likely to report mental health problems compared to their urban counterparts. Lifetime history of TBI with LOC was associated with increased risk of binge drinking, heavy drinking, depression diagnoses, and poor general mental health, regardless of location of living.

Similar to previous research in the general population [21,22], we found that rurality was associated with a lower risk of binge drinking and heavy drinking among those with lifetime history of TBI with LOC. The existing evidence from the 2001–2002 National Epidemiologic Survey on Alcohol and Related Conditions suggests that those living in rural areas in the Midwest who did not report abstaining from drinking were at similar or lower risk compared to those living in urban areas of the Midwest, although they were at a greater risk for an alcohol disorder and exceeding the daily limits for drinking (e.g., binge drinking) compared to those living in suburban areas of the Midwest [23]. These findings suggest that living in a rural area may continue to act as a protective factor against unhealthy alcohol use among those with a lifetime history of TBI with LOC. While differences in alcohol use behaviors may vary by location of living due to social, cultural, legal, and health system characteristics [21,23], further research is necessary to determine which of these factors may interact with location of living in contributing to unhealthy alcohol use among those with a lifetime history of TBI with LOC.

Our results suggested that living in a rural area was not associated with an increased risk of a depression diagnosis or general poor mental health after adjusting for age, gender, race, and lifetime history of TBI with LOC. Using the 2009–2011 National Survey of Drug Use and Health, Breslau and colleagues [14] found that the prevalence of major depression was higher in small metro and semi-rural areas than in large metro areas, even after adjusting for demographics. Similar to alcohol use, cultural, social, and economic factors all interact to impact mental health, which may explain the differences found by Breslau and colleagues [14] as there is greater variation among these factors in a national sample versus our sample from a single state. However, our findings were consistent with some previous findings in the general population where the contribution of demographics and health characteristics to the prevalence of mental health problems may not differ between rural and urban populations [13,15]. For example, females typically report greater rates of mood disorders and mental health issues than males, regardless of whether they are living in a rural or urban area [13,15]. Although not at an increased risk, those living in rural areas may have limited access to quality health care, including mental health care, compared to those in urban areas [24,25]. While little data are available comparing access to follow-up care or brain-injury specialists in rural versus urban areas following TBI [26], given the evidence supporting that increased access to trauma care positively impacts TBI outcomes in rural areas [11], future research and health policies should address access to mental health care services following a TBI among rural residents.

Finally, as expected, regardless of location of living, lifetime history of TBI with LOC was associated with an increased risk of binge drinking and heavy drinking, supporting the prior findings addressing this relationship among Ohioans [5] and North Carolinians [6]. Our results showed that those with a lifetime history of TBI with LOC were more than twice as likely to be diagnosed with a depressive disorder compared to those without lifetime history of TBI with LOC, regardless of location of living, strengthening the need to address mental health care services following TBI [5,6]. Similar to earlier studies’ findings [5,7], we also found that lifetime history of TBI with LOC was associated with reporting ≥2 days and ≥14 days of poor mental health in the past 30 days in rural and urban residents. Furthermore, the non-overlapping CIs suggested that the risk for ≥14 days of poor mental health was even greater than the risk for ≥2 days of poor mental health among those with a lifetime history of TBI with LOC. The long-term adverse behavioral health outcomes following TBI are likely in part caused by physical and/or functional damage to the brain during the injury, negatively impacting the executive functions (e.g., decision making, impulse control, emotional expression) [4,27] and/or neural pathways responsible for dopamine production and uptake [4,28]. This damage may be particularly important for TBIs that occur during childhood as they may interrupt the normal development patterns, exacerbating these issues later in life [5,28,29]. Future research should address the possible influences of the nature and characteristics of TBIs, including the number of TBIs experienced, severity of TBIs, age of first TBI, and recency of last TBI, on these long-term behavioral health outcomes.

The primary limitation of this study is the use of a cross-sectional design, which prevented us from addressing causal relationships between rurality, lifetime history of TBI with LOC, unhealthy alcohol use, and mental health problems. Additionally, data on the location of the TBI (i.e., if the injury occurred in an urban or rural area) and length or changes in current location of living (i.e., how long the respondent has been living in their current location) are not available. Therefore, it is possible that the TBI with LOC occurred in a different location of living or that respondents have migrated between rural and urban areas. Future studies should employ longitudinal study designs to assess these relationships. Second, this study relied on self-report data. While the measures used for lifetime history of TBI, unhealthy alcohol use, and mental health problems have been well validated, as with any self-report, it is possible that the observed associations could be inaccurate due to recall bias or social desirability. Third, the study used a dichotomous definition of location of living (rural vs. urban) based on the Office of Management and Budget definition [18] and did not measure availability of, access to, or utilization of mental health care. Other measures of rurality, such as rural-urban continuum codes (RUCC) [30] or rural-urban commuting areas (RUCA) [31], use more categories to reflect the nuances of rurality. Further research could address if the definition of rurality used impacts the present findings and the availability, accessibility, and utilization of mental health care among those with a lifetime history of TBI. Finally, this study did not assess the nature and characteristics of TBI, including the number of TBIs, severity of TBI, age of first TBI, and recency of last TBI. Given the evidence from previous studies [4,5,6,7] suggesting these factors may be associated with various behavioral health outcomes, future research should address how rurality may moderate these relationships.

## 5. Conclusions

In the present study, those living in a rural area were at a reduced risk for unhealthy alcohol use compared to those in urban areas, but rurality did not modify the relationships between lifetime history of TBI with LOC and behavioral health issues. Given those living in rural areas may have limited access to quality health care, including mental health care, compared to those in urban areas, future research and health policies should address access to mental health services following a TBI among rural residents. This study provided additional evidence of the associations between lifetime history of TBI with LOC and unhealthy alcohol use and mental health problems among adults living in rural areas. These findings support the need for TBI screenings as part of mental health history intake evaluations and behavioral health screenings.

## Figures and Tables

**Table 1:** Demographics and lifetime history of TBI (original and weighted samples) ^1^.

	Unweighted *n*	Weighted *n (%)*
Overall	16,941	7,578,787 (100.0)
Survey Year		
2016	7128	3,165,459 (41.8)
2017	3128	1,409,229 (18.6)
2018	3328	1,493,316 (19.7)
2019	3357	1,510,783 (19.9)
Gender		
Male	6939	3,631,381 (47.9)
Female	10,002	3,947,407 (52.1)
Age Group		
Age 18 to 44	3596	3,262,709 (43.1)
Age 45 to 64	6441	2,613,487 (34.5)
Age 65 or older	6904	1,702,592 (22.5)
Race		
White	14,902	6,150,936 (81.2)
Black	1077	838,535 (11.1)
Other ^2^	731	485,575 (6.4)
Unknown	231	103,741 (1.4)
Location of Living		
Urban	10,285	5,841,243 (77.1)
Rural	6656	1,739,800 (22.9)
Lifetime TBI with LOC		
No	14,213	6,296,117 (83.1)
Yes	2728	1,282,670 (16.9)

^1^ Data were expressed as weighted number (*n*) and percent prevalence (%). Totals do not add up to 100% due to rounding. The weighted frequencies and prevalence percentages were estimated using the statewide weighting variable of individuals with the weighted survey procedures in SAS. ^2^ Other includes Asians, Native Hawaiians, other Pacific Islanders, American Indians, or Alaska Natives.

**Table 2:** Associations of lifetime history of TBI and demographics with unhealthy alcohol use and mental health among rural and urban respondents.

	Binge Drinking	Heavy Drinking	Diagnosis of Depressive Disorder	Poor Mental Health for 2 or More Days in the Past 30 Days	Poor Mental Health for 14 or More Days in the Past 30 Days
Overall	Weighted *n* (%)	Weighted *n* (%)	Weighted *n* (%)	Weighted *n* (%)	Weighted *n* (%)
	1,282,668 (16.9)	496,670 (6.6)	1,525,094 (20.1)	2,534,643 (33.4)	1,077,455 (14.2)
Rural Respondents					
Lifetime TBI with LOC					
No	198,497 (13.9) ***	69,848 (4.9) ***	247,732 (17.3) ***	420,919 (29.4) ***	169,687 (11.9) ***
Yes	66,170 (21.4)	27,960 (9.1)	104,592 (34.0)	137,317 (44.6)	81,238 (26.4)
Gender					
Male	177,743(22.2) ***	59,964 (7.5) ***	110,466 (13.8) ***	196,692 (24.5) ***	92,177 (11.5) ***
Female	86,924 (9.3)	37,844 (4.0)	241,858 (25.9)	361,545 (38.6)	158,748 (17.0)
Age Group					
Age 18 to 44	152,000 (22.2) ***	44,827 (6.5) ***	151,525 (22.1) ***	289,862 (42.3) ***	120,820 (17.6) ***
Age 45 to 64	91,502 (14.4)	42,023 (6.6)	144,421 (22.8)	191,476 (30.2)	97,831 (15.4)
Age 65 or older	21,165 (5.1)	10,977 (2.6)	56,378 (13.5)	76,899 (18.4)	32,273 (7.7)
Race					
White	244,035 (15.3)	88,485 (5.5)	310,704 (19.5) *	502,321 (31.5)	223,966 (14.0)
Black	4995 (14.3)	3090 (8.9)	10713 (30.8)	13515 (38.8)	6453 (18.5)
Other ^2^	11,440 (13.7)	4789 (5.7)	25,854 (30.9)	34,191 (40.8)	15,718(18.8)
Unknown	4197 (18.7)	1445 (6.4)	5053 (22.5)	8209 (36.5)	4788 (21.3)
Urban Respondents					
Lifetime TBI with LOC					
No	791,389 (16.3) ***	302,128 (6.2) ***	844,991 (17.4) ***	1,526,352 (31.4) ***	572,771 (11.8) ***
Yes	227,231 (22.3)	97,112 (10.0)	329,875 (33.8)	448,993 (46.1)	253,253 (26.0)
Gender					
Male	651,204 (23.0) ***	228,871 (8.1) ***	454,907 (16.1) ***	852,336 (30.1) ***	352,671 (12.5) **
Female	367,416 (12.2)	170,369 (5.7)	719,960 (23.9)	1,123,010 (37.3)	473,353 (15.7)
Age Group					
Age 18 to 44	670,642 (26.0) ***	212,272 (8.3) ***	569,622 (22.1) ***	1,120,693 (43.5) ***	442,573 (17.2) ***
Age 45 to 64	288,534 (14.6)	143,285 (7.2)	432,508 (21.8)	618,373 (31.2)	303,918 (15.3)
Age 65 or older	59,444 (4.6)	43,683 (3.4)	172,737 (13.5)	236,281 (18.4)	79,534 (6.2)
Race					
White	833,266 (18.3) **	347,733 (7.6) ***	928,677 (20.4)	1,529,442 (33.6) *	627,991 (13.8) *
Black	99,059 (12.3)	18,128 (2.3)	133,699 (16.6)	261,180 (32.5)	106,650 (13.3)
Other ^2^	66,519 (16.6)	24,013 (6.0)	96,127 (23.9)	164,247 (40.9)	78,449 (19.5)
Unknown	19,776 (24.3)	9366 (11.5)	16,365 (20.1)	20,449 (25.2)	12,935 (15.9)

* *p* < 0.05, ** *p* < 0.01, *** *p* < 0.001; TBI—traumatic brain injury; LOC—loss of consciousness.

**Table 3:** Unadjusted and adjusted odds ratios (OR) for unhealthy alcohol use outcomes.

	Binge Drinking		Heavy Drinking	
	Unadjusted OR (95% CI)	Adjusted OR (95% CI) ^1^	Unadjusted OR (95% CI)	Adjusted OR (95% CI) ^1^
Living in a rural area				
Yes	0.85 (0.74–0.97)	0.84 (0.73–0.97)	0.81 (0.67–0.99)	0.75 (0.62–0.92)
No	Reference	Reference	Reference	Reference
Lifetime TBI with LOC				
Yes	1.59 (1.35–1.87)	1.36 (1.14–1.61)	1.72 (1.38–2.16)	1.52 (1.20–1.91)
	Reference	Reference	Reference	Reference
Gender				
Male	0.44 (0.38–0.51)	0.46 (0.40–0.53)	0.64 (0.53–0.78)	0.70 (0.58–0.85)
Female	Reference	Reference	Reference	Reference
Age Group				
Age 18 to 44	6.78 (5.57–8.24)	6.87 (5.63–8.37)	2.58 (2.02–3.28)	2.59 (2.03–3.30)
Age 45 to 64	3.42 (2.82–4.14)	3.33 (2.74–4.04)	2.30 (1.83–2.88)	2.24 (1.78–2.81)
Age 65 or older	Reference	Reference	Reference	Reference
Race				
Non-White	0.67 (0.50–0.89)	0.58 (0.43–0.78)	0.34 (0.23–0.51)	0.32 (0.21–0.48)
White	Reference	Reference	Reference	Reference

^1^ Adjusted for gender, age, race, location of living, and lifetime history of TBI with LOC; TBI—traumatic brain injury; LOC—loss of consciousness; the interaction between location of living and lifetime history of TBI with LOC was not significant for any outcome and thus was not included in the adjusted model.

**Table 4:** Unadjusted and adjusted odds ratios (OR) for depression and poor mental health days outcomes.

	Diagnosis of Depressive Disorder		Poor Mental Health for 2 or More Days in the Past 30 Days		Poor Mental Health for 14 or More Days in the Past 30 Days	
	Unadjusted OR (95% CI)	Adjusted OR (95% CI) ^1^	Unadjusted OR (95% CI)	Adjusted OR (95% CI) ^1^	Unadjusted OR (95% CI)	Adjusted OR (95% CI) ^1^
Living in a rural area						
Yes	1.01 (0.9–1.14)	0.98 (0.87–1.10)	0.93 (0.84–1.03)	0.93 (0.84–1.04)	1.03 (0.89–1.18)	1.03 (0.89–1.19)
No	Reference	Reference	Reference	Reference	Reference	Reference
Lifetime TBI with LOC						
Yes	2.44 (2.12–2.8)	2.61 (2.26–3.01)	1.88 (1.65–2.14)	1.96 (1.72–2.24)	2.64 (2.25–3.09)	2.68 (2.28–3.16)
No	Reference	Reference	Reference	Reference	Reference	Reference
Gender						
Male	1.75 (1.54–1.98)	1.99 (1.75–2.26)	1.48 (1.33–1.65)	1.68 (1.50–1.87)	1.37 (1.18–1.59)	1.57 (1.35–1.82)
Female	Reference	Reference	Reference	Reference	Reference	Reference
Age Group						
Age 18 to 44	1.82 (1.58–2.1)	1.82 (1.57–2.11)	3.38 (2.98–3.82)	3.43 (3.02–3.90)	2.97 (2.49–3.54)	2.88 (2.41–3.44)
Age 45 to 64	1.82 (1.61–2.07)	1.78 (1.57–2.03)	1.99 (1.78–2.23)	1.97 (1.76–2.21)	2.58 (2.19–3.04)	2.48 (2.10–2.92)
Age 65 or older	Reference	Reference	Reference	Reference	Reference	Reference
Race						
Non-White	0.83 (0.65–1.05)	0.82 (0.64–1.06)	0.99 (0.82–1.20)	0.90 (0.73–1.10)	0.97 (0.75–1.25)	0.97 (0.74-1.27)
White	Reference	Reference	Reference	Reference	Reference	Reference

^1^ Adjusted for gender, age, race, location of living, and lifetime history of TBI with LOC; TBI—traumatic brain injury; LOC—loss of consciousness; the interaction between location of living and lifetime history of TBI with LOC was not significant for any outcome and thus was not included in the adjusted model.

## Data Availability

The data that support the findings of this study are available from the Ohio Department of Public Health, but restrictions apply to the availability of these data, which were used under license for the current study and, therefore, are not publicly available. Data are, however, available from the authors upon reasonable request and with permission of the Ohio Department of Public Health.

## References

[1] Peterson A.B., Xu L., Daugherty J., Breiding M.J. (2019). Surveillance Report of Traumatic Brain Injury-Related Emergency Department Visits, Hospitalizations, and Deaths. United States, 2014.

[2] Gooch C.L., Pracht E., Borenstein A.R. (2017). The Burden of Neurological Disease in the United States: A Summary Report and Call to Action. Ann. Neurol..

[3] Menon D.K., Schwab K., Wright D.W., Maas A.I. (2010). The Demographics and Clinical Assessment Working Group of the International and Interagency Initiative toward Common Data Elements for Research on Traumatic Brain Injury and Psychological Health. Position statement: Definition of traumatic brain injury. Arch. Phys. Med. Rehabil..

[4] Corrigan J.D. (2021). Traumatic Brain Injury and Treatment of Behavioral Health Conditions. Psychiatr. Serv..

[5] Corrigan J.D., Hagemeyer A.N., Weil Z.M., Sullivan L., Shi J., Bogner J., Yang J. (2020). Is Pediatric Traumatic Brain Injury Associated with Adult Alcohol Misuse?. J. Neurotrauma.

[6] Waltzman D., Daugherty J., Sarmiento K., Proescholdbell S. (2021). Lifetime History of Traumatic Brain Injury with Loss of Consciousness and the Likelihood for Lifetime Depression and Risk Behaviors: 2017 BRFSS North Carolina. J. Head Trauma Rehabil..

[7] Whiteneck G.G., Cuthbert J.P., Corrigan J.D., Bogner J.A. (2016). Risk of Negative Outcomes After Traumatic Brain Injury: A Statewide Population-Based Survey. J. Head Trauma Rehabil..

[8] Leonhard M.J., Wright D.A., Fu R., Lehrfeld D.P., Carlson K.F. (2015). Urban/rural disparities in Oregon pediatric traumatic brain injury. Inj. Epidemiol..

[9] Brown J.B., Kheng M., Carney N.A., Rubiano A.M., Puyana J.C. (2019). Geographical disparity and traumatic brain injury in America: Rural areas suffer poorer outcomes. J. Neurosci. Rural Pract..

[10] Gabella B., Hoffman R.E., Marine W.W., Stallones L. (1997). Urban and Rural Traumatic Brain Injuries in Colorado. Ann. Epidemiol..

[11] Tiesman H., Young T., Torner J.C., McMahon M., Peek-Asa C., Fiedler J. (2007). Effects of a rural trauma system on traumatic brain injuries. J. Neurotrauma.

[12] Simpson G., McRae P., Hallab L., Strettles B. (2016). Vocational outcomes from the New South Wales Brain Injury Rehabilitation Program: A multi-centre study. Brain Inj..

[13] Probst J.C., Laditka S.B., Moore C.G., Harun N., Powell M.P., Baxley E.G. (2006). Rural-Urban Differences in Depression Prevalence: Implications for Family Medicine. Health Serv. Res..

[14] Breslau J., Marshall G.N., Pincus H.A., Brown R.A. (2014). Are mental disorders more common in urban than rural areas of the United States?. J. Psychiatry Res..

[15] Weaver A., Himle J.A., Taylor R.J., Matusko N.N., Abelson J.M. (2015). Urban vs Rural Residence and the Prevalence of Depression and Mood Disorder Among African American Women and Non-Hispanic White Women. JAMA Psychiatry.

[16] Centers for Disease Control and Prevention Behavioral Risk Factor Surveillance System: BRFSS Survey Data and Documentation. https://www.cdc.gov/brfss/data_documentation/index.htm.

[17] Corrigan J.D., Bogner J. (2007). Initial reliability and validity of the Ohio State University TBI Identification Method. J. Head Trauma Rehabil..

[18] U.S Department of Health and Human Services, Health Resources and Services Administration, Office of Management and Budget Defining Rural Population. https://www.census.gov/programs-surveys/metro-micro.html.

[19] National Institutes of Health, National Institute on Alcohol Abuse and Alcoholism (2005). Helping Patients Who Drink Too Much. https://pubs.niaaa.nih.gov/publications/practitioner/cliniciansguide2005/guide.pdf.

[20] Centers for Disease Control and Prevention, National Center for Chronic Disease Prevention and Health Promotion Division of Population Health BRFSS Prevalence & Trends Data. https://www.cdc.gov/brfss/brfssprevalence/index.html.

[21] Dixon M.A., Chartier K.G. (2016). Alcohol Use Patterns Among Urban and Rural Residents. Alcohol Res. Curr. Rev..

[22] Matthews K.A., Croft J.B., Liu Y., Lu H., Kanny D., Wheaton A.G., Kettel Khan L., Caraballo R.S., Eke P.I., Giles W.H. (2017). Health-Related Behaviors by Urban-Rural County Classification—United States, 2013. Morb. Mortal. Wkly. Rep..

[23] Borders T.F., Booth B.M. (2007). Rural, suburban, and urban variations in alcohol consumption in the United States: Findings from the National Epidemiologic Survey on Alcohol and Related Conditions. J. Rural Health.

[24] Douthit N., Kiv S., Dwolatzky T., Biswas S. (2015). Exposing some important barriers to health care access in the rural USA. Public Health.

[25] Substance Abuse and Mental Health Services Administration (2016). Rural Behavioral Health: Telehealth Challenges and Opportunities. Breif.

[26] Gardner J., Sexton K.W., Taylor J., Beck W., Kimbrough M.K., Davis B., Bhavaraju A., Karim S., Porter A. (2018). Defining severe traumatic brain injury readmission rates and reasons in a rural state. Trauma Surg. Acute Care Open.

[27] Courville C. (1950). Pathology of the Central Nervous System.

[28] Karelina K., Gaier K.R., Weil Z.M. (2017). Traumatic brain injuries during development disrupt dopaminergic signaling. Exp. Neurol..

[29] Bigler E.D., Maxwell W.L. (2012). Neuropathology of mild traumatic brain injury: Relationship to neuroimaging findings. Brain Imaging Behav..

[30] Economic Research Service—US Department of Agriculture Rural-Urban Continuum Codes. https://www.ers.usda.gov/data-products/rural-urban-continuum-codes.aspx.

[31] Economic Research Service—US Department of Agriculture Rural-Urban Commuting Area Codes. https://www.ers.usda.gov/data-products/rural-urban-commuting-area-codes.aspx.

